# Advance care planning preferences in Chinese nursing home residents: results from two cross-sectional studies in Hong Kong and Taiwan

**DOI:** 10.1186/s12904-021-00820-4

**Published:** 2021-08-03

**Authors:** Xinyi Xu, Shu-Wen Tu, Chia-Chin Lin

**Affiliations:** 1 School of Nursing, Li Ka Shing Faculty of Medicine, The University of Hong Kong, Hong Kong , Hong Kong; 2 Taipei Veterans General Hospital Yuli Branch, Taipei, Taiwan; 3 Alice Ho Miu Ling Nethersole Charity Foundation, Hong Kong, Hong Kong; 4 School of Nursing, College of Nursing, Taipei Medical University, Taipei, Taiwan

**Keywords:** Advance directive, Advance care planning, End-of-life care, Nursing home, Long-term care

## Abstract

**Background:**

The proportion of hospital deaths has declined in the past few decades, while the proportions of nursing home deaths have increased. This trend of increasing deaths in long-term care facilities underlines the importance of improving end-of-life care provisions in these settings to meet individual preferences and needs. Under these circumstances, a comprehensive understanding of end-of-life care preferences in local nursing home residents can help healthcare professionals and policymakers develop strategies to increase the advance directive completion rate and quality of care. This study aimed to explore and compare advance directive and end-of-life care preferences of nursing home residents in Hong Kong and Taiwan.

**Methods:**

A structured questionnaire was developed by the research team to investigate advance directive and end-of-life care preferences in older Chinese nursing home residents. Nursing home residents with frail or pre-frail status and over the age of 64 were invited to participate in the study, and information on demographics, functional status, advance directive experiences, and end-of-life care expectations was collected through questionnaire interviews.

**Results:**

A total of 325 eligible participants from 32 facilities completed the survey, including 238 older residents in Hong Kong and 87 in Taiwan. A significantly lower proportion of the Hong Kong residents had completed an advance directive compared with the Taiwanese (3 vs. 13%, *p* = 0.001). Among participants who did not have an advance directive, 46% of the Taiwanese participants said they would consider completing one in the future, compared with 20% of the Hong Kong participants (*p* < 0.001). A total of 79% of the Hong Kong participants and 80% of the Taiwanese participants responded that prolonging life in the given hypothetical dying scenario was “not important” (*p* = 0.76). Only 14% of participants in Hong Kong and 18% of participants in Taiwan reported prior occurrence of end-of-life care discussions with family members or health professionals (*p* = 0.37).

**Conclusions:**

This paper adds evidence in support of improving end-of-life communication and the advance directive completion rate in nursing homes in Hong Kong and Taiwan. Further research is necessary to explore cross-cultural differences in end-of-life preferences and its applications in predicting decision-making and the quality of end-of-life care.

**Supplementary Information:**

The online version contains supplementary material available at 10.1186/s12904-021-00820-4.

## Background

In the past two decades, global life expectancy has increased by 7.7 years, which is projected to increase by an additional 4.5 years by 2050 [1]. The rapid growth of the elderly population and increased prevalence of chronic diseases not only drive the demand for long-term care but also influence the place of death. Population-based studies have shown that while the proportion of hospital deaths has declined in the past few decades, the proportions of care and nursing home deaths have increased [2–4]. This trend of increasing deaths in long-term care facilities underlines the importance of improving end-of-life care provisions in these settings to meet individual preferences and needs.

Advance care planning (ACP) is a process of communication that enables individuals to express preferences for future medical care, which may involve the creation of a written document known as an *advance directive* (AD) [5]. Several literature reviews have found that having an AD and ACP are associated with positive patient-centered outcomes, including improved quality of end-of-life care, reduced life-sustaining treatments, and increased palliative care utilization [6, 7]. Furthermore, several randomized controlled studies have demonstrated that ADs and ACP programs could reduce hospitalizations and medical costs in nursing home residents while improving the quality of care [8, 9].

Despite growing findings that favor the use of ADs, AD completion rates remain low worldwide, with a high variation in different countries. For instance, the AD completion rate among the general population is merely 0.5% in Hong Kong and 2.6% in Taiwan, compared with 10–14% among Chinese Americans [10–12]. The low AD completion rates in these two places indicate room for improvement in both regions.

Hong Kong and Taiwan, two developed regions with predominately Han Chinese populations, are both challenged by rapidly aging populations as well as increasing demands for residential elderly care services [13, 14]. Under these circumstances, a comprehensive understanding of end-of-life care preferences in local nursing home residents can help healthcare professionals and policymakers develop strategies to increase the AD completion rate and quality of care. In this paper, we aimed to investigate and compare AD and end-of-life preferences of nursing home residents in Hong Kong and Taiwan.

## Methods

Two cross-sectional studies were carried out, one in Taiwan and one in Hong Kong. A structured questionnaire was developed by the research team to investigate end-of-life preferences in older Chinese nursing home residents. Nursing home is defined as a facility providing long-term functional support and nursing care for elders who require assistance with ADLs and having identified health needs [15]. AD is defined as any documentations of advance decisions to refuse life-sustaining treatments when the person can no longer make decisions and terminally ill. In both settings, AD signing requires two witnesses, one of whom must be a medical practitioner. Individuals are encouraged to discuss with family member before making AD in Hong Kong, and individuals in Taiwan are required an ACP consultation process before making AD [16, 17].

This questionnaire was piloted with a sample of 10 participants in Taiwan to test for its feasibility and was subsequently revised, with excellent content validity as rated by six local experts in Taiwan (Content Validity Index on Average; CVI/AVR = 0.988). The first cross-sectional study was conducted in five nursing homes in northern Taiwan from December 2015 to November 2016. The second study was conducted in 27 nursing homes in Hong Kong from May 2019 to December 2019. The questionnaire used in the first study was revised further for the study in Hong Kong based on two rounds of local expert review and a pilot of 12 participants (CVI/AVR = 0.87). With core questions regarding end-of-life preference unchanged, compared with the Taiwan version, the revised questionnaire included additional secondary measurements, such as the McGill Quality of Life tool as well as a section on place of dying. To compare the results of these two studies, only demographic information and core items on end-of-life care preferences and advance directive completion were included in the analysis. Nursing homes in both regions were recruited through purposive sampling by sending out mail invitations, and face-to-face interview were conducted by trained research assistants.

The research was performed in accordance with the Declaration of Helsinki; Ethics approval was obtained from the institutional review board of Taipei Medical University and the institutional review boards of the University of Hong Kong/the Hong Kong West Cluster of the Hong Kong Hospital Authority, and informed consent was obtained from each participant. The STROBE statement was used as a guideline in preparation of this manuscript. All methods were performed in accordance with the relevant guidelines and regulations.

### Study 1—Taiwan

#### Participants and procedures

The inclusion criteria for participation were as follows: i) at least 65 years of age and ii.) frail or pre-frail Chinese in nursing homes who could speak and understand either Mandarin or Taiwanese [18]. Residents with cognitive impairments (scoring 5 or below on the abbreviated mental test) or communication problems were excluded. Ethics approval for data collection was obtained from the institutional review board of Taipei Medical University, and written informed consent was obtained from each participant.

#### Questionnaire

The questionnaire comprised four parts: (A) demographics and functional status as measured by Activities of Daily Living (ADL); (B) healthcare experiences and attitude; (C) expectations of end-of-life care; (D) AD experience.

### Study 2—Hong Kong

#### Participants and procedures

The inclusion criteria for participation were as follows: i) at least 65 years of age; ii) frail or pre-frail Chinese in nursing homes who could speak and understand either Cantonese or Mandarin [18]. Residents with cognitive impairments (scoring 5 or below on the abbreviated mental test) or communication problems were excluded. This study was approved by the institutional review boards of the University of Hong Kong and the Hong Kong West Cluster of the Hong Kong Hospital Authority. An information sheet was presented to each participant, and oral consent was sought.

#### Questionnaire

The questionnaire was comprised seven parts: (A) demographics and functional status as measured through ADL; (B) healthcare experiences and attitude; (C) expectations of end-of-life care; (D) ACP experience and attitude; (E) AD awareness, experience, and attitude; (F) ideal terminal/death environment (preferences on places of care and eventual death); and (G) quality of life. A detailed description of the questionnaire has been published elsewhere [19].

### Analysis

Study population characteristics and end-of-life preferences were assessed using descriptive statistics, and differences between the participants of the two regions were assessed using Pearson’s chi-square tests. All statistical analyses were performed using SPSS software (version 25.0; IBM Corp., Armonk, NY, USA), and a *p*-value of < 0.05 was considered significant.

### Patient and public involvement (PPI) statement

Older residents were not directly involved in the development of the surveys as the local nursing home management are motivated to protect the residents and may hesitate to participate in the research without the formal ethical approval. The surveys were developed after consultation with local experts who work closely with the target population. The study findings will be disseminated in plain language in nursing homes and to general public to strengthen the development of end-of-life care in Hong Kong and Taiwan.

## Results

A total of 325 eligible participants completed either version of the survey, including 238 older residents in Hong Kong and 87 in Taiwan. The recruited participants’ mean age was 85 years, and 55% were female. Around 59 percent of them identified themselves as being affiliated with a religion, and 35% had no formal education. One-fifth of the participants had severe to total dependence (0–60 on a 0–100 scale), as assessed using the Barthel Index of ADL [20]. As to educational level, sex, emergency room visit(s) in the past year, and relative/friend(s) who had passed in the past 2 years, data were similar between the two groups, while age, marital status, religious belief, ADL scores, and length of nursing home stay were significantly different. The demographic characteristics of the participants are shown in Table 1.

**Table 1:** Demographic characteristics of nursing home residents in Hong Kong and Taiwan

	**Hong Kong**	**Taiwan**	***P* value**	**Missing**
	***n* = 238**	***n* = 87**		***n* = 325**
	**n (%)**	**n (%)**		**n (%)**
Age				
65–74	43 (18.1%)	35 (40.2%)	.000	8 (2.5%)
75–84	63 (26.5%)	28 (32.2%)		
> 85	124 (52.1%)	24 (27.6%)		
Mean	87.43	78.26		
Sex				
Male	102 (42.9%)	43 (49.4%)	.292	
Female	136 (57.1%)	44 (50.6%)		
Educational level				
No formal education	86 (36.1%)	28 (32.2%)	.464	7 (2.2%)
Primary	89 (37.4%)	41 (47.1%)		
Secondary	40 (16.8%)	11 (12.6%)		
Tertiary or above	16 (6.7%)	7 (8%)		
Marital status				
Single	19 (8%)	19 (21.8%)	.002	
Married	59 (24.8%)	21 (24.1%)		
Widowed/Divorced/Other	160 (67.2%)	47 (54%)		
Religion				
Christianity	39 (16.4%)	12 (13.8%)	.000	2 (0.6%)
Buddhism/Taoism	52 (21.8%)	36 (41.4%)		
Islam	38 (16%)	1 (1.1%)		
Other	14 (5.9%)	0 (0%)		
Unaffiliated	95 (39.9%)	36 (41.4%)		
Activities of daily living				
61–80	181 (76.1%)	79 (90.8%)	.001	1 (0.3%)
31–60	37 (15.5%)	1 (1.15%)		
< 30	20 (8.4%)	6 (6.9%)		
Length of stay in nursing home				
≤ 2 years	116 (48.7%)	51 (58.6%)	.039	22 (6.8%)
2–5 years	47 (19.7%)	21 (24.1%)		
> 5 years	53 (22.3%)	15 (17.2%)		
Emergency room visit in the past year				
Yes	95 (40%)	27 (31%)	.117	4 (1.2%)
No	139 (58.4%)	60 (69%)		
Relatives or friends passing in the past 2 years				
Yes	84 (35.3%)	28 (32.2%)	.551	3 (0.9%)
No	151 (63.4%)	59 (67.8%)		

### AD completion and consideration

The prevalence of ADs across the participants is shown in Table 2. A total of 13% of the participants in Taiwan had made ADs compared with merely 3% in Hong Kong (*p* = 0.001). The top reasons for AD signing in Taiwan were “preventing unnecessary suffering during resuscitation” (*n* = 4), “documentation of personal medical preference” (*n* = 3), and “wish for peaceful and dignified death” (*n* = 2). Similarly, the top reasons for AD signing in Hong Kong were “documentation of personal medical preference” (*n* = 4) and “wish for peaceful and dignified death” (*n* = 3).

**Table 2:** Advance directive usage and end-of-life preferences of nursing home residents in Hong Kong and Taiwan

	**Hong Kong**	**Taiwan**	***P* value**	**Missing**
	***n* = 238**	***n* = 87**		***n* = 325**
	**n (%)**	**n (%)**		**n (%)**
In a hypothetical dying scenario of “becoming seriously ill but with no cure available,” do you think prolonging life is important?				
Yes/Not sure	50 (21%)	17 (20%)	.76	1 (0.3%)
No	187 (79%)	70 (80%)		
Have you ever participated in end-of-life care discussions with family members or health professionals?				
Yes	34 (14%)	16 (18%)	.37	1 (0.3%)
No	203 (85%)	71 (82%)		
Do you have an advance directive?				
Yes	7 (3%)	11 (13%)	.001	4 (1.2%)
No	227 (95%)	76 (87%)		
Would you consider completing an advance directive in the future?				
Yes	45 (20%)	35 (46%)	< 0.001	4 (1.2%)
No	182 (80%)	41 (54%)		

Around 46% of the Taiwanese participants who did not have an AD indicated they would consider completing an AD in the future. Taiwanese participants who did not have an AD nor considered completing one reported that their top reasons for not considering were “unnecessary at the moment” (*n* = 31) and “health professions can make future medical decisions” (*n* = 6). However, a majority (80%) of the Hong Kong participants said they would not consider AD signing as a means to document their end-of-life care preferences, and the main reasons for this were “unnecessary at the moment” (*n* = 89) and “health professionals can make future medical decisions” (*n* = 55). The difference in AD consideration was statistically significant between the participants of the two regions (*p* < 0.001).

### The importance of prolonging life at the end-of-life

In a hypothetical dying scenario of “becoming seriously ill but with no cure available,” participants were asked whether they believed prolonging life would be important. The attitudes toward prolonging life at the end-of-life were similar in the two regions. A total of 79% of the Hong Kong participants and 80% of the Taiwanese participants responded that prolonging life in the given hypothetical dying scenario was “not important” (*p* = 0.76).

### End-of-life care discussion

The majority of the participants in both regions had never engaged in discussions regarding preferred end-of-life care with their family members or any medical professionals, such as physicians, nurses, or social workers. Only 14% of participants in Hong Kong and 18% of participants in Taiwan reported prior occurrence of end-of-life care discussions (*p* = 0.37).

## Discussion

This paper explored AD and end-of-life preferences among Chinese nursing home residents in Hong Kong and Taiwan. Two cross-sectional studies demonstrated that the AD completion rate was higher among nursing home residents in Taiwan than in Hong Kong, even though most participants in both groups believed prolonging life at the end-of-life stage was not important. Additionally, high proportions of residents in both regions had never had end-of-life care discussions with their family or any medical professionals.

It was anticipated that a higher proportion of Taiwanese participants would have an AD compared with Hong Kong participants, as the AD completion rate has been reported to be five times higher in the general population in Taiwan than in Hong Kong [10, 12]. A total of 13% of Taiwanese nursing home residents in our study had an AD, which is comparable to the rates identified in the existing literature [18, 19]. Lo (2010) found that among 201 residents in southern Taiwanese nursing homes, 16.4% had ADs [21, 22]. Lo [21] found that among 201 residents in southern Taiwanese nursing homes, 16.4% had ADs, while in a retrospective study, Tsai [22] found that 12.9% of northern Taiwanese nursing home residents had ADs. Even though the prevalence of ADs among nursing home residents in Hong Kong has not been previously reported, Chu et al. [23] found a high preference of 88% for ADs in this population. Contrastingly, we identified a low AD completion rate of 3% in our Hong Kong sample as well as a relatively low preference for ADs (19%). The questionnaire design could have played a role in these discrepancies. In Chu’s [23] study, participants were asked whether they agreed that it would be beneficial to have an AD to express their preferences; in our studies, participants were directly asked if they would consider completing an AD in the future after an explanation of what it was. The distinction between acknowledging the potential benefits of having an AD and the actual consideration of completing one requires further investigation. In contrast, the willingness to complete an AD was significantly higher in the Taiwanese sample (40%). The legal status of ADs in the two regions might explain the differences in AD completion rates and AD considerations.

The development of end-of-life and palliative care germinated around the early 1980s in both Hong Kong and Taiwan [24, 25]. However, the legal status of ADs and the quality of end-of-life care differ between the two regions today. In Hong Kong, end-of-life wishes can be documented in advance under the common law, even though the use of ADs has not been legislated. In cases of conflict with other statutory provisions, ADs will be superseded by exiting legislation [26, 27]. In Taiwan, the Hospice Palliative Care Act was passed in 2000, guaranteeing the right to dignified death in terminally ill patients, and the National Health Insurance began to provide full coverage for inpatient palliative care. In 2015, the Taiwanese legislature passed the Patient Right to Autonomy Act, which is the first law in Asia aimed at protecting a patient’s right to autonomy, including exercising the right to refuse medical treatments through ADs [28]. After the passing of the Hospice Palliative Care Act, Taiwan experienced an increase in supportive care use in different intensive care units for stroke patients and a decline in the use of intensive procedures [29]. In addition, population-based studies have found that the implementation of palliative care policies in Taiwan was associated with improved palliative care utilization in connection with cancer, dementia, chronic obstructive pulmonary disease, and stroke [29–31].

According to the 2015 Quality of Death Index, Taiwan ranked first in Asia with regards to quality of death and sixth among 80 countries and regions, with Hong Kong ranking twenty-second [32]. The lack of legally backed ADs, comprehensive palliative care policies, and universal health insurance coverage of palliative care in Hong Kong seems to widen the gap in the quality of palliative care in this region. The significant differences between Taiwan and Hong Kong in legislation, policy, and insurance of palliative care also offer potential explanations for the low AD completion rate and low AD preference in Hong Kong nursing home residents.

Despite the differences in AD completion rates and AD consideration, the experience in end-of-life discussions and attitudes towards prolonging life at the end of care were similar in Hong Kong and Taiwan. Consistent with previous studies, the majority of the nursing home residents in our studies valued comfort over prolonging the length of life in a hypothetical end-of-life scenario (79 vs. 80%, *p* = 0.76) [23, 33–35]. Yet, only around one in five residents had engaged in end-of-life care discussions. This outcome is not surprising because traditionally, death is viewed as a taboo in Chinese culture, and discussions on death and dying are avoided for fear of invoking bad luck [36]. Furthermore, low awareness and lack of available information on AD and ACP in these populations might have also contributed to the low frequencies of end-of-life discussions [23, 37]. Several systematic reviews have shown that structured communication tools may increase the frequency of discussions about and completion of ADs, and the application of such tools should be tailored to local needs. Culturally sensitive ACP and AD interventions should be designed for Chinese nursing home residents to improve communication around medical decision-making and end-of-life care [38]. For example, indirect communication strategies and a family-centered approach might be more appropriate in this population [39].

The group of residents who reported having an AD was small; nevertheless, it would be interesting to use multivariable statistical analysis to examine the factors associated with having an AD. In the adjusted model, residing in Taiwan, having secondary or above education, and participation in end-of-life care discussions were significantly associated with having an AD (Additional file 1). The analysis is limited by the sparse data, the results must be interpreted with caution.

In conclusion, this study adds evidence in support of improving end-of-life communication and the AD completion rate in nursing homes in Hong Kong and Taiwan. To meet the needs of the Chinese nursing home resident population, further development of an end-of-life care model that integrates family-centered care, culturally sensitive communication strategies, and care preference documentation is recommended. In addition, the Hong Kong Special Administrative Region Government should develop policies and legislation to facilitate the implementation of AD usage, especially in nursing home residents. Further research is also needed to explore cross-cultural differences in end-of-life preferences and its applications in predicting decision-making and the quality of end-of-life care.

## Limitations

The two studies have several limitations. First, the cross-sectional design of these studies cannot conclude causality. Second, participants with communication barriers (i.e., deafness) were excluded due to the nature of the data collection method. Third, the sample size was relatively small, and studies with larger sample sizes are needed to validate the conclusions. Fourth, there is a difference in age distribution which might be due to difference in life expectancy and general characteristics among Hong Kong and Taiwan nursing home residents. Lastly, information on the response rate and characteristics of the nonrespondents were not collected, which might lead to biased prevalence estimates and selection bias. The study results should be interpreted with caution.

## Conclusion

This paper shows that nursing home residents in Hong Kong had a significant lower completion rate of AD and willingness to complete one in the future compared to residents in Taiwan, and the differences in legal status, policy, and insurance coverage of AD and end-of-life care between two regions might have influences their AD preference. Our results also demonstrate the similarity of two regions in desire for quality of death and a lack of end-of-life communication with loved ones or healthcare professionals. This paper highlights that health policy and administration system are important factors in ACP preferences in nursing home residents.

## Supplementary Information

**Additional file 1.** Association between advance directive and demographic and other factors.

## Data Availability

Data are available upon reasonable request. Full data set available on request via corresponding author (cclin@hku.hk).

## Abbreviations

ACP: Advance care planning
AD: Advance directive
ADL: Activities of daily living
