# Supplementary material for: Advance care planning preferences in Chinese nursing home residents: results from two cross-sectional studies in Hong Kong and Taiwan
Source: BMC Palliat Care. 2021 Aug 3;20:123. doi: 10.1186/s12904-021-00820-4 (PMC8336386; doi:10.1186/s12904-021-00820-4)
Supplement: Supplementary file 1 — Additional file 1. Association between advance directive and demographic and other factors. [file 12904_2021_820_MOESM1_ESM.docx]

Association between advance directive and demographic and other factors

| **Factor** | **Unadjusted OR (95% CI)** | **Adjusted OR (95% CI)** |
| --- | --- | --- |
| Age | 0.95 (0.90 – 1.01) | 1.00 (0.94 – 1.07) |
| Sex |  |  |
| Female | ref | ref |
| Male | 2.62 (0.96 – 7.18) | 2.26 (0.63 – 8.07) |
| Region |  |  |
| Taiwan | ref | ref |
| Hong Kong | 0.21 (0.08 – 0.57) | 0.13 (0.04 – 0.50) |
| Education |  |  |
| No formal education/primary education | ref | ref |
| Secondary or above | 4.62 (1.75 – 12.2) | 7.50 (2.09 – 26.94) |
| Martial status |  |  |
| Single/Widowed/Divorced | ref | ref |
| Married | 0.87 (0.28 - 2.72) | 0.56 (0.14 – 2.27) |
| Religion |  |  |
| Unaffiliated | ref | ref |
| Affiliated | 1.38 (0.51 – 3.78) | 0.69 (0.21 – 2.32) |
| Activities of daily living |  |  |
| 61–80 | ref | ref |
| ≤60 | 0.23 (0.03 – 1.74) | 0.42 (0.04 – 4.42) |
| Length of stay in nursing home |  |  |
| ≤2 years | ref | ref |
| 2–5 years | 2.27 (0.73 – 7.03) | 3.53 (0.88 – 14.16) |
| >5 years | 1.35 (0.42 – 4.39) | 2.77 (0.63 – 12.10) |
| Emergency room visit in the past year |  |  |
| No | ref | ref |
| Yes | 1.05 (0.40 – 2.78) | 1.36 (0.40 – 4.64) |
| Relatives or friends passing in the past two years |  |  |
| No | ref | ref |
| Yes | 1.98 (0.76 – 5.14) | 2.43 (0.79 – 7.42) |
| In a hypothetical dying scenario of “becoming seriously ill but with no cure available,” do you think prolonging life is important? |  |  |
| No | ref | ref |
| Yes/Not sure | 0.75 (0.21 – 2.66) | 0.87 (0.20 – 3.70) |
| Have you ever participated in end-of-life care discussions with family members or health professionals? |  |  |
| No | ref | ref |
| Yes | 6.39 (2.40 – 17.04) | 11.06 (3.06 – 39.97) |
